# Bee Venom Acupuncture in Traditional Korean Medicine: A Review of Clinical Practice Guidelines

**DOI:** 10.3390/toxins17040158

**Published:** 2025-03-22

**Authors:** Minjung Park, Seungwon Shin

**Affiliations:** 1 College of Korean Medicine, Gachon University, Seongnam 13120, Republic of Korea; mjimage@hanmail.net; 2 College of Korean Medicine, Sangji University, Wonju 26339, Republic of Korea

**Keywords:** bee venom, acupuncture, pharmacopuncture, clinical practice guideline, traditional Korean medicine

## Abstract

Bee venom acupuncture (BVA) is used in traditional Korean medicine (TKM) for various diseases, but its evaluation within clinical practice guidelines (CPGs) has not been comprehensively reviewed. This study aimed to review TKM-CPGs to characterize the range of conditions for which BVA is recommended, summarize the level of evidence and recommendation grades, and assess the factors influencing the grades. Eighteen TKM-CPGs, including 30 BVA-related recommendations, were identified. Data on targeted diseases/symptoms, treatment protocols, evidence levels, and recommendation grades were extracted. The CPGs recommended BVA for musculoskeletal and neurological disorders in standalone or combined therapy. Most of the evidence for BVA recommendations was evaluated with low to moderate levels based on randomized controlled trials. The grades of recommendations were mostly B or C, indicating that BVA is advisable or potentially beneficial. Although the CPGs offer some guidance on treatment protocols for BVA, there remains a lack of detailed specifications, and we need to conduct additional research to provide evidence. Also, the heterogeneity of recommendations across different CPGs presents a challenge in establishing consistent clinical guidelines. Future research should focus on generating high-quality evidence and standardizing treatment regimens to support more robust recommendations for BVA in TKM clinical practice.

## 1. Introduction

Bee venom (BV), a complex mixture of biologically active substances secreted by honeybees (*Apis mellifera*), has been used for centuries in traditional medicine for its therapeutic properties [1]. It contains peptides such as melittin, apamin, adolapin, and mast cell degranulating peptide, along with various enzymes, notably phospholipase A_2_ and hyaluronidase. Melittin exhibits potent anti-inflammatory, cytolytic, and antimicrobial properties, primarily by disrupting cellular membranes, while apamin has significant neurological effects by selectively blocking calcium-dependent potassium channels. Additionally, phospholipase A_2_ contributes anti-inflammatory, immune-modulatory, and antiviral effects by hydrolyzing membrane phospholipids, and hyaluronidase facilitates venom diffusion and enhances tissue permeability by degrading extracellular matrix components, collectively broadening BV’s therapeutic potential [2,3]. This natural compound has been applied in various forms, including acupuncture and topical applications, to treat multiple conditions such as arthritis, rheumatism, back pain, and skin diseases [4]. Modern research has revealed that BV possesses potent anti-inflammatory and analgesic properties [5,6], supporting its potential application in treating various inflammatory conditions.

In clinical practice, bee venom acupuncture (BVA) is employed across a spectrum of pain conditions [7], primarily within musculoskeletal and neuropathic contexts. For musculoskeletal pain, BVA has demonstrated effectiveness in alleviating low back pain, shoulder pain, and osteoarthritis [4,8,9]. Beyond its use in musculoskeletal disorders, BVA has shown promising effects for neurological and oncological conditions. A systematic review indicated the potential benefits of BVA in managing post-stroke shoulder pain [10]. Furthermore, BV and its primary component, melittin (MEL), have shown anticancer effects against various malignancies, including hepatocellular carcinoma, prostate cancer, melanoma, and lung cancer, by inhibiting cancer cell growth, inducing apoptosis, and suppressing tumor metastasis [11,12]. These diverse applications of BV highlight potential as a therapeutic agent across multiple medical domains [13].

Clinical practice guidelines (CPGs) are systematically developed statements to assist practitioners and patients in making decisions about appropriate health care for specific clinical circumstances [14]. These guidelines play a crucial role in evidence-based medicine by synthesizing the best available evidence and expert consensus to provide recommendations for clinical practice [15]. CPGs serve as essential tools for standardizing treatment approaches, improving patient outcomes, and ensuring evidence-based decision-making in clinical practice. They aim to improve the quality of patient care, reduce inappropriate variation in practice, promote efficient use of resources, and identify knowledge gaps that require further research [16].

The development of CPGs in traditional Korean medicine (TKM) has evolved significantly. While initial TKM-CPG development was primarily led by academic societies, subsequent efforts, mainly through national research and development projects funded by the Ministry of Health and Welfare (MoHW) in the Republic of Korea, have emphasized validated evidence conforming to international standards. These initiatives have resulted in developing numerous TKM-CPGs across various clinical problems, with globally standardized methodologies including Grading of Recommendations, Assessment, Development and Evaluations (GRADE) and Appraisal of Guidelines for Research and Evaluation (AGREE) II assessments, promoting evidence-based medical services in TKM [17].

Despite growing evidence supporting BVA’s therapeutic potential, systematic analyses of its clinical application remain limited. Recent systematic reviews and meta-analyses have reported the effects of BVA in diverse diseases, including motor function for Parkinson’s disease [18], post-stroke pain [19], whiplash injury [20], and frozen shoulder [21]. TKM-CPGs incorporate BVA recommendations for many diseases, which vary in specific treatment regimens, such as dosage, frequency, and acupoint selection.

Therefore, this study aimed to review the BVA-related recommendations within TKM-CPGs, outline how BVA has been recommended for treatment across a wide array of clinical conditions, summarize the level of evidence and recommendation grades of BVA recommendations, and assess the factors influencing these grades in order to provide clinicians with insights into the appropriate use of BVA and identify areas for future research.

## 2. General Characteristics of BVA-Related Recommendations in TKM-CPGs

**Figure 1:**
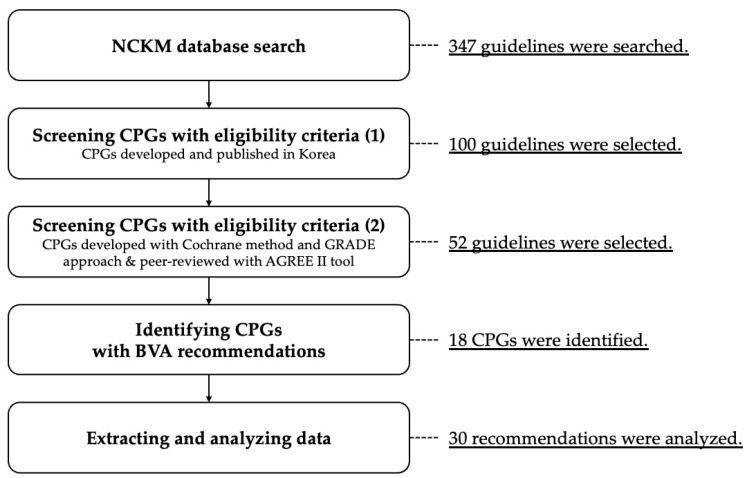
Study workflow to identify recommendations for bee venom acupuncture therapy in clinical practice guidelines of traditional Korean medicine. Abbreviations. AGREE, Appraisal of Guidelines for Research and Evaluation; CPG, clinical practice guideline; GRADE, Grading of Recommendations, Assessment, Development and Evaluations; NCKM, National Clearinghouse for Korean Medicine.

A search of the National Clearinghouse for Korean Medicine (NCKM, nikom.or.kr/nckm) database was conducted in January 2025 and identified a total of 347 CPGs. Among these, 100 were guidelines developed in Korea. Further assessment revealed that 52 of these 100 guidelines were developed with verified quality control processes, following standardized manuals [22,23]. Of these 52 guidelines, 18 included recommendations for BVA therapy. We finally verified the 30 specific BVA recommendations in the selected 18 CPGs (Figure 1).

The 18 CPGs included in this review encompassed a wide range of clinical conditions (Table 1). Musculoskeletal disorders were the most frequently represented, including chronic low back pain [24], cervical pain [25], shoulder pain [26], knee osteoarthritis [27], lumbar herniated intervertebral disc [28], ankle sprain [29], carpal tunnel syndrome [30], rheumatoid arthritis [31], degenerative arthritis of hip and phalangeal joints [32], and temporomandibular joint disorder [33]. Furthermore, CPGs for the postoperative syndromes following spinal, shoulder, or knee surgery [34] and traffic accident injury specified with whiplash-associated disorders I and II [35] were also considered as musculoskeletal conditions. The CPGs addressing neurological diseases were also included, such as stroke [36] and facial nerve palsy [37]. Additionally, there were the CPGs covering other diseases, including tension-type headache [38], cancer-accompanying symptoms [39], prostatic hypertrophy [40], and gout [41]. These 18 CPGs recommended BVA as a standalone treatment or in combination with other therapeutic methods. The details on combined therapies are summarized in the subsequent section for BVA treatment regimens.

Most of the CPGs relied on primary studies conducted in Korea. For example, the guidelines for ankle sprain [29], carpal tunnel syndrome [30], cervical pain [25], chronic low back pain [24], degenerative arthritis [32], and gout [41] exclusively used Korean studies. Some CPGs incorporated studies from both Korea and China, such as facial nerve palsy [37], rheumatoid arthritis [31], and temporomandibular joint disorder [33]. Only the CPG for knee osteoarthritis included a multinational randomized controlled trial (RCT) [27].

Age-related conditions for BVA therapy were recommended, with most guidelines applicable to general adults without specifying age ranges. However, the guideline for traffic accident injury specifically addressed adults aged 19–70 years old for BVA therapy [35]. Disease or symptom specifications of BVA recommendations were also presented. For acute conditions, BVA was recommended for acute ankle sprain [29] and acute gout [41]. Chronic conditions included chronic ankle sprain [29], chronic and nonspecific low back pain [24], and chronic tension-type headache [38]. The CPG for post-surgical care recommended BVA for patients in rehabilitation periods after lumbar surgery, total knee arthroplasty, and rotator cuff operations [34]. Neurological conditions such as facial palsy (including idiopathic cases with or without post-auricular pain) [37] and stroke-related symptoms (motor disability, shoulder pain, and spasticity) [36] were also addressed. Other specifications for BVA included cancer-related pain [39], degenerative arthritis of the hip and phalangeal joints [32], and benign prostatic hyperplasia without acute urinary retention [40]. Gender-specific recommendations were absent in the included CPGs. Most guidelines did not differentiate between males and females.

BVA therapy was recommended within the broader category of pharmacopuncture therapy in a few CPGs, such as chronic low back pain [24], facial nerve palsy [37], postoperative syndrome [34], temporomandibular joint disorder [33], tension-type headache [38], and traffic accident injury [35].

The complete recommendations outlined in each disease-specific guideline are presented in Table S1.

## 3. Regimen of BVA Therapy Recommended in TKM-CPGs

Several CPGs recommended BVA as a standalone therapy for specific conditions. This monotherapeutic approach was suggested for chronic low back pain [24], degenerative arthritis [32], facial nerve palsy [37], gout [41], knee osteoarthritis [27], prostatic hypertrophy [40], rheumatoid arthritis [31], and temporomandibular joint disorder [33]. BVA was also recommended as part of a combined therapeutic approach, incorporating other modalities alongside BVA. The accompanied treatments often included acupuncture therapy (AT) [25,29,32], electroacupuncture (EA) [30], usual care (UC) [28,31,34,35,36,37], analgesics [39], physical therapy (PT) [26], and herbal medicine (HM) [38] (Table 1).

The selected CPGs offered specific guidance on the BVA regimens for clinical practice. Key parameters included the acupoints, the concentration and dosage of BVA administered, and the frequency and duration of treatment sessions. Table 2 summarizes the details of the BVA regimen.

Many different acupoints for BVA were recommended across CPGs, depending on the targeted condition. These included local acupoints near the site of pain or dysfunction (called Ashi points) and distal acupoints on the meridians associated with the affected area. The data show that Ashi points were recommended [28,31,32,34,35,36,39,41], and these were often in conjunction with acupoints of the stomach (ST) [29,33,34,37,39], bladder (BL) [24,29,34,40], and gallbladder (GB) [24,29,32,34,35,36,37] meridians. The CPGs for carpal tunnel syndrome [30], cervical pain [25], knee osteoarthritis [27], shoulder pain [26], and tension-type headache [38] did not address specific acupoints for BVA treatment.

The included recommendations exhibited similarities in BVA concentration and dosage. For example, the CPGs for ankle sprain [29], chronic low back pain [24], and degenerative arthritis [32] recommended concentrations ranging from 3000:1 to 20,000:1. In terms of dosage, several CPGs advised administering 0.05 to 0.2 cc with an increasing dose when necessary [24,29,32,36]. The frequency for BVA treatments was typically around two to three sessions per week [24,32,36]. Notably, only one CPG for stroke specified the treatment duration of 2 to 4 weeks [36]. There were many BVA recommendations which did not specify concentration, dose, frequency, or duration [26,30,31,33,35,37,38,39,40,41].

Most CPGs, except for tension-type headache [38] and prostatic hypertrophy [40], explicitly recommended skin hypersensitivity testing to ensure the safe administration of BVA.

## 4. Level of Evidence for BVA Therapy in TKM-CPGs

Many CPGs recommended BVA based on RCTs, implying an effort to incorporate higher-quality evidence. However, there were CPGs recommending BVA based on non-randomized studies (NRSs), such as degenerative arthritis of the hip joint [32], gout [41], and postoperative syndrome after lumbar surgery [34]. The reliance on NRS may reflect a lack of RCTs. Meanwhile, the recommendations for degenerative arthritis of the phalangeal joint [32], postoperative syndrome of total knee arthroplasty and rotator cuff surgery [34], prostatic hypertrophy [40], and traffic accident injury [35] did not present any supporting clinical studies due to a lack of appropriate research. As a result, the level of evidence for these recommendations was either not assessed [32,40] or rated at the lowest level (“Very low”) [34,35]. There were no recommendations with high-level evidence (Table 1).

Table 3 summarizes the level of evidence and its relevant information on BVA recommendations presented in the CPGs with solely RCTs. Among the recommendations for standalone BVA therapy, the level of evidence was found to be either moderate or low. For chronic low back pain [24] and temporomandibular joint disorder [33], the level was moderate, while for facial nerve palsy [37], knee osteoarthritis [27], and rheumatoid arthritis [31], it was low. For combined therapy recommendations, the level of evidence ranged from moderate to very low. Moderate levels of evidence were found in lumbar herniated intervertebral disc [28], traffic accident injury [35], shoulder pain [26], and cervical pain [25], while low levels were found in cancer-accompanying symptoms [39], shoulder pain or motor disability due to stroke [36], rheumatoid arthritis [31], ankle sprain [29], and idiopathic facial palsy with post-auricular pain [37].

The sample size was also a notable concern. While some CPGs, such as lumbar herniated intervertebral disc [28] and knee osteoarthritis [27], included RCTs with relatively large sample sizes (*n* = 382 and *n* = 367, respectively), many other recommendations relied on RCTs with smaller sample sizes. These smaller sample sizes could limit the statistical power to detect true effects and may contribute to imprecision in the effect estimates.

The GRADE assessment criteria revealed common factors that led to downgrading the level of evidence. Imprecision, related to small sample sizes and wide confidence intervals, was the most frequent factor [25,26,27,28,29,30,31,33,35,36,37,38]. RoB also contributed to downgrading [24,27,29,30,31,36,37,38,39], indicating methodological limitations of the primary RCTs. Inconsistency was noted in some cases [29,37,39], suggesting heterogeneity among study findings. Indirectness was only cited as a reason for downgrading in tension-type headache [38]. Publication bias was not mentioned in any of the guidelines.

## 5. Grade of Recommendation for BVA Therapy in TKM-CPGs

Table 4 summarizes the grade of recommendation for BVA therapy across the included TKM-CPGs. The grades generally reflect the strength of the recommendation, ranging from Grade A (strongly endorsed) to Grade D (not recommended) [17,22,23].

Overall, the grades of recommendation for BVA varied across conditions. A significant proportion of the recommendations were graded as B or C, indicating that BVA is generally considered advisable or potentially beneficial in specific clinical conditions. Only one recommendation for temporomandibular joint disorder reached Grade A [33], suggesting BVA as a first-line treatment in most patients. A few CPGs did not provide a formal grade, instead offering “Good Practice Points (GPP)”, indicating consensus support by the expert panel [32,40,41]. There were no recommendations graded level D.

The CPGs considered multiple factors when assigning recommendation grades, including potential benefits, harms, level of evidence, availability in clinical practice, medical costs, and patients’ preferences, following the standardized manuals [17,22,23].

All CPGs assessed the potential benefits and the level of evidence of BVA treatment. Most guidelines reported positive effects, noting significant improvements in pain relief and functional outcomes for conditions.

The CPGs, except for gout [41], also mentioned potential harms. The CPGs for knee osteoarthritis [27] and neck pain due to traffic accident injury [35] performed the harm assessment with the selected primary studies. A larger group of CPGs relied on pre-existing safety reports, literature reviews, or professional consensus to assess harm [24,25,26,28,29,31,32,33,34,35,37,39]. However, some recommendations for carpal tunnel syndrome [30], stroke [36], and facial nerve palsy [37] did not draw firm conclusions about the harm of BVA due to insufficient data, while those for benign prostatic hyperplasia [40] and tension-type headache [38] mentioned harm without presenting specific reasons.

Fourteen CPGs assessed the availability of BVA in clinical practice. Preceding reports for the utilization statistics of BVA were employed in many recommendations [28,31,32,33,35,39,40,41], while some proposed the wide availability of BVA based on professional consensus [24,26,29,30,34,37,38]. Some recommendations for ankle sprain [29], cervical pain [25], facial nerve palsy [37], knee osteoarthritis [27], and stroke [36] did not assess availability.

The assessment of medical costs was less common and less conclusive. The CPG for temporomandibular joint disorder conducted an economic evaluation [33], while most CPGs drew inconclusive decisions about costs due to data insufficiency [24,30,34,36,39]. Regarding the patients’ preference for BVA, there were only two CPGs addressing this. The recommendation for cancer-related pain assessed the patients’ preference with professionals’ opinions [39], while prostatic hypertrophy mentioned it without any concrete reasons [40].

## 6. Discussion and Future Directions

This study aimed to investigate clinical recommendations related to BVA within CPGs in TKM. Our comprehensive review identified 18 CPGs containing 30 specific BVA recommendations across various diseases, primarily musculoskeletal and neurological disorders. The CPGs recommended BVA as a standalone therapy or in combination with other TKM modalities (AT, EA, or HM). The acupoints used for BVA included a high proportion of Ashi points but varied depending on the targeted disease. The regimen specifications varied across CPGs, with a dose of 0.05 to 0.2 cc, concentration ranging from 3000:1 to 20,000:1, treatment frequency (typically around two to three sessions per week), and duration, while others still lacked specific treatment protocols. Most CPGs recommended BVA therapy based on RCTs to demonstrate higher-quality evidence. However, the level of evidence varied, with most falling into the moderate to low levels. No high-quality evidence was found. The grades of recommendation for BVA also varied, with most being graded as B or C, indicating that BVA is generally considered advisable or potentially beneficial in clinical practice, but not strongly endorsed as a first-line treatment. Notably, the assessment of factors such as potential benefits, harms, availability in clinical practice, medical costs, and patient preferences varied considerably across the guidelines, with only a few CPGs providing comprehensive evaluations of all these aspects.

Our review reveals a potential gap between the clinical practice where BVA is utilized and the conditions explicitly addressed in the CPGs. Laboratory experiments have identified the therapeutic effects of BVA for immunological and neurological diseases, including autoimmune diseases and Parkinson’s diseases [13]. There is also clinical evidence suggesting that BVA may be beneficial in several areas not covered by these CPGs, such as chemotherapy-induced peripheral neuropathy of cancer patients [42], bone fracture [43], or Parkinson’s disease [44]. Furthermore, the CPGs often lacked specific treatment protocols tailored to factors such as age, gender, disease severity, or associated symptoms. Even for conditions included in the CPGs, the treatment protocols, including acupoint selection criteria, dosage, and treatment duration, were frequently unspecified. This absence of detailed guidance may hinder the implementation of BVA in clinical practice, as clinicians may struggle to adapt the recommendations to individual patient needs. Future CPG development should focus on expanding the scope of conditions covered and incorporating more granular treatment protocols to address these gaps.

The moderate to low levels of evidence supporting BVA recommendations pose challenges for clinicians seeking to adopt these guidelines. The level-downgrading due to the RoB of the primary studies may stem from methodological limitations such as inadequate randomization, lack of blinding, or incomplete outcome reporting. To address these issues, future research must prioritize globally standardized trial designs adhering to frameworks like Consolidated Standards of Reporting Trials (CONSORT) [45] and Standards for Reporting Interventions in Clinical Trials of Acupuncture (STRICTA) [46]. The imprecision observed in several recommendations for BVA therapy implies the need for confirmatory RCTs with sufficient sample sizes. Most current studies lack statistical power to detect clinically meaningful differences, limiting their utility in guiding practice. Furthermore, the lack of standardization in venom dosage and administration protocols remains a significant barrier to high-quality BVA RCTs. Additionally, concerns regarding patient safety, including potential adverse reactions and anaphylaxis risks, necessitate rigorous safety assessments and standardized monitoring protocols in future trials.

The predominance of B or C grades in BVA recommendations can be attributed to the primary studies’ methodological limitations. Deciding recommendation grades was strongly influenced by the quality and consistency of RCT findings, which often exhibited methodological deficits such as small sample sizes, inadequate blinding, and variability in treatment protocols. Also, many CPGs lacked assessments for harm, availability in practice, cost-effectiveness, and patient preference. As a result, the combination of methodological weaknesses in RCTs and insufficient evaluation of other factors led to a conservative interpretation of BVA’s role, restricting its recommendation to an advisable or potentially beneficial treatment rather than a strongly endorsed first-line therapy.

The limited assessment of harms, cost-effectiveness, and patient preferences hinders the ability to generate more stringent recommendation grades. It has been reported that BV exhibits notable cytotoxic and genotoxic effects. High concentrations of BV have been shown to induce significant cytotoxicity, leading to cell death in human lymphocytes, which implies potential risks to normal cells upon exposure [47]. Furthermore, BV has demonstrated genotoxic effects, including DNA strand breaks and chromosomal aberrations in human peripheral blood lymphocytes, highlighting the necessity of caution regarding dosage and clinical applications [48]. A Korean survey of 468 pharmacopuncture patients found that patients administered BVA therapy experienced more severe adverse events than other pharmacopuncture therapies [49]. Specifically, localized side effects, such as pruritus, erythema, edema, and pain at the injection site, and systemic adverse events, such as headache, dizziness, nausea, hyperventilation, and chest pain, have been documented [6,49]. In rare cases, severe allergic reactions such as urticaria, angioedema, and anaphylaxis have also been reported [50]. These emphasize the need for careful patient screening before treatment. A previous meta-analysis highlighted that only 10% of BVA cases documented pre-treatment allergy testing [51]. It is remarkable that most CPGs in this review recommended pre-treatment skin testing to mitigate allergic risks.

Cost-effectiveness or cost-utility evaluations remain a critical gap. Although BVA showed effectiveness for chronic neck pain when combined with non-steroidal anti-inflammatory drugs (NSAIDs) [52], an objective assessment is needed to determine the incremental effectiveness or utility gained relative to the additional costs incurred compared to NSAID monotherapy. Unlike AT or EA, BVA is not covered by national health insurance in Korea, and we need evidence to evaluate its potential benefits and value.

Patient preference data were also sparse. A survey conducted in Saudi Arabia revealed that rheumatoid arthritis patients showed a more favorable attitude towards BVA than those with other chronic diseases [53]. An objective assessment of patient preferences can play a crucial role in weighing a treatment’s overall benefits and harms.

This study has several limitations. First, this review did not include any guidelines from other countries that may also practice BVA, resulting in a geographically imbalanced dataset. Second, this study reviewed the CPGs that have already been published. Therefore, the diseases covered in this study may not represent the full spectrum of conditions for which BVA is used in clinical practice. New guidelines for different conditions are developing but might not yet be published [17,54]. Third, the AGREE II tool assessment for the included CPGs was not performed since we only included the CPGs for which the assessments had already been performed. Fourth, we could not compare BVA with other alternative therapies due to the lack of comprehensive reviews evaluating the evidence levels and recommendation grades of those interventions across CPGs. Meanwhile, there was a review of CPGs for cupping therapy (CT) in Korea [55]. Similar to BVA, most CT recommendations were graded as B or C, with evidence levels ranging from low to moderate due to concerns about methodological limitations in primary studies. Both therapies faced downgrading of evidence due to risk of bias and imprecision, reflecting the challenges in conducting high-quality randomized controlled trials. Further research is required to systematically compare the clinical evidence of these treatments within the framework of CPGs.

Despite these limitations, this study also has several strengths. First, it provides a comprehensive review of the evidence base for BVA, allowing for an overview of when and how it can be applied to various diseases. Second, by comparing the level of evidence and recommendation grades across different CPGs, we could offer concrete guidance for future BVA research.

In conclusion, this review reveals that BVA is recommended primarily for musculoskeletal and neurological disorders in standalone or combined therapy, mostly with low to moderate levels of evidence. Grades of recommendation were typically B or C, which indicates that BVA is generally advisable or potentially beneficial. While the CPGs offer some guidance on treatment protocols, there remains a lack of detailed specifications. For practitioners, these findings suggest that while BVA may offer potential benefits, its clinical application should be approached with caution. Clinicians should carefully consider individual patient conditions and weigh potential benefits and risks to make decisions in practice.

Future research should prioritize high-quality RCTs, standardized treatment protocols, and comprehensive evaluation of all relevant factors to strengthen the evidence base and inform more robust CPGs for BVA in TKM. Efforts should be made to establish standardized venom dosage guidelines, ensure consistent methodology in clinical trials, and enhance safety assessments to mitigate potential risks associated with BVA. Also, future CPGs should incorporate more rigorous evaluations of patient preferences, treatment availability, and cost-effectiveness to support more comprehensive and clinically applicable recommendations.

## 7. Methods

The present review employed a structured approach to identify, assess, and summarize information from TKM-CPGs recommending BVA. We adopted a methodological framework similar to a previous study that reviewed TKM-CPGs and their recommendations for cupping therapy [55].

### 7.1. Identification and Selection of TKM-CPGs for BVA

This review focused solely on CPGs developed and published in Korea, ensuring that all included guidelines were created following standardized methodologies and assessment tools. To capture pertinent CPGs, we searched the guideline library of NCKM, an authoritative repository funded by MoHW of Korea. No specific language restrictions were applied. CPGs were identified by utilizing the filtering options available in the NCKM database. Then, the following inclusion criteria were applied to select eligible CPGs for this review:

- CPGs developed and published in Korea by January 2025.
- CPGs developed following standardized manuals [22,23], which incorporate systematic reviews based on the Cochrane method, evidence level and recommendation grade assessment using the GRADE approach, and external peer review using the AGREE II tool.
- CPGs including BVA recommendations, either as a standalone treatment or in combination with other therapeutic methods. We included CPGs explicitly addressing BVA, as well as those recommending BVA as part of a general pharmacopuncture therapy. However, CPGs that only mention pharmacopuncture without explicitly including BVA were excluded.

### 7.2. Data Extraction

#### 7.2.1. General Characteristics of the Included TKM-CPGs

A structured data extraction protocol was employed to gather relevant information systematically from the selected CPGs. The extracted data encompassed the targeted disease, the specified conditions (age, sex, or clinical subtypes) for BVA recommendations, the treatment modality of BVA in each recommendation (monotherapy or polytherapy), and the number, design, and geographical origin of the primary studies included in each recommendation for BVA.

#### 7.2.2. Regimen Specifications of BVA

Specific details of the BVA regimen outlined in the CPGs were also extracted. This included the acupoints for BVA therapy and the concentration, the injected dose per treatment, the frequency of treatment sessions, and the duration of the treatment course.

#### 7.2.3. Evidence Appraisal

Data regarding the quantity and quality of evidence underlying each recommendation were extracted. This process focused on identifying the level of evidence assigned to each recommendation and the factors that led to its designation. According to the GRADE approach, the factors influencing the level of evidence were the risk of bias (RoB), imprecision, inconsistency, indirectness, and publication bias [22,23,55].

Each level of evidence (high, moderate, low, very low) has the following meaning: the evidence is evaluated with a high level when there is robust confidence that the true effect is close to the estimated effect. It is assessed with a moderate level when further research may potentially affect the estimated effect. It is evaluated with a low level when there is limited confidence in the effect estimate, and the true effect could be substantially different. Finally, it is evaluated with a very-low level when the true effect diverges significantly from the estimate. In this case, additional research is very likely to change the actual effect estimate [22,23,55].

#### 7.2.4. Grade of Recommendation and Its Rationale

The grade of each recommendation and supporting information were also extracted. Factors that CPG working groups considered to grade each recommendation of BVA were also abstracted, including the potential benefit and harm, the level of evidence, the availability in clinical practice, medical costs, and patient preferences of BVA [22,23,55].

Grades of recommendations are assigned based on the manuals. The grades generally reflect the strength of the recommendation: Grade A indicates that the treatment is strongly endorsed for implementation in almost all clinical practices. Grade B is assigned when the treatment is advisable in most clinical situations. Grade C denotes that the therapy has potential benefits in some but not all clinical practice. Grade D is presented when the treatment is not recommended in most clinical practices due to insufficient evidence of benefit or potential harm [22,23,55].

### 7.3. Data Analysis and Presentation

The extracted data were synthesized and summarized using tables and figures to provide a comprehensive overview of the evidence base for BVA in TKM-CPGs. The review focused on identifying patterns, trends, and gaps in the evidence and practice for BVA.

## Figures and Tables

**Table 1:** Clinical practice guidelines recommending bee venom acupuncture therapy in traditional Korean medicine.

CPG for	Targeted for	Treatment Modality	Primary Studies (No. and Origination)	
Ankle sprain [29]	Adults, acute ankle sprain	Combined therapy with AT	4 RCTs	Korea
	Adults, chronic ankle sprain	Combined therapy with AT	1 NRS	Korea
Cancer-accompanying symptoms [39]	Cancer-related pain	Combined therapy with analgesics	3 RCTs	China
Carpal tunnel syndrome [30]	Adults	Combined therapy with EA	1 RCT	Korea
Cervical pain [25]	Adults	Combined therapy with AT	2 RCTs	Korea
Chronic low back pain [24]	Adults, chronic and nonspecific low back pain	Standalone therapy *	3 RCTs	Korea
Degenerative arthritis (hip and hand) [32]	Hip joint	Standalone therapy	1 NRS	Korea
	Hip joint	Combined therapy with AT and EA	0	
	Phalangeal joint	Standalone therapy	0	
Facial nerve palsy [37]	Facial palsy with delayed treatment	Standalone therapy	2 RCTs	China
	Idiopathic facial palsy	Combined therapy with TKM UC	2 RCTs	Korea
	Idiopathic facial palsy with post-auricular pain	Combined therapy with TKM UC *	3 RCTs	Korea
Gout [41]	Acute gout	Standalone therapy *	1 NRS	Korea
Knee osteoarthritis [27]	NS	Standalone therapy	1 RCT	Multinational
Lumbar herniated intervertebral disk [28]	Adults	Combined therapy with TKM UC	8 RCTs	China (2) Korea (6)
Postoperative syndrome [34]	Patients with persistent or recurrent pain or in rehabilitation period after lumbar surgery	Combined therapy with TKM UC *	7 NRSs	Korea
	Patients in rehabilitation period after total knee arthroplasty	Combined therapy with TKM UC *	0	
	Patients in rehabilitation period after rotator cuff operation	Combined therapy with TKM UC *	0	
Prostatic hypertrophy [40]	Benign prostatic hyperplasia without acute urinary retention	Standalone therapy	0	
Rheumatoid arthritis [31]	Adults	Standalone therapy	3 RCTs	China (2) Korea (1)
	Adults of no symptom improvement with either TKM or WM treatments alone	Combined therapy with WM UC	3 RCTs	China
Shoulder pain [26]	Adults	Combined therapy with PT	2 RCTs	Korea
Stroke [36]	Motor disability with stroke	Combined therapy with TKM UC	3 RCTs	Korea
	Shoulder pain with stroke	Combined therapy with TKM UC	5 RCTs	Korea
	Spasticity with stroke	Combined therapy with TKM UC	2 RCTs	Korea
Temporomandibular joint disorder [33]	NS	Standalone therapy *	2 RCTs	China (1) Korea (1)
Tension-type headache [38]	Chronic tension-type headache	Combined therapy with HM *	1 RCT	China
Traffic accident injury (whiplash-associated disorders I, II) [35]	Adults (19–70 yrs old), low back and neck pain	Standalone therapy *	0	
	Adults (19–70 yrs old), neck pain	Combined therapy with TKM UC *	3 RCTs	Korea

* BVA therapy is recommended within a scope of pharmacopuncture therapy. Abbreviations: AT, acupuncture therapy; BVA, bee venom acupuncture; CPG, clinical practice guideline; EA, electroacupuncture; HM, herbal medicine; NRS, nonrandomized studies; NS, not specified; PT, physical therapy; RCT, randomized controlled trial; TKM, traditional Korean medicine; UC, usual care; WM, western medicine.

**Table 2:** Regimen of bee venom acupuncture therapy in clinical practice guidelines of traditional Korean medicine.

CPG for	Acupoints for BVA	Regimen
Ankle sprain [29]	BL60, BL62, GB39, GB40, GB41, KI3, KI6, LR4, SP5, SP6, SP9, ST36	(concentration) 20,000:1~3000:1 (dose) 0.05~1 cc; increase when necessary
Cancer-accompanying symptoms [39]	Ashi and Back-Shu points, SP6, ST36	NS
Carpal tunnel syndrome [30]	NS	NS
Cervical pain [25]	NS	(dose) NS; but caution advised
Chronic low back pain * [24]	BL23, BL24, BL25, GB30, GV3, GV4, GV5	(concentration) 20,000:1 (dose) 0.2 cc; gradual increase (max 1.2 cc) (frequency) 2 sessions/week
Degenerative arthritis * [32]	(Hip joint) Ashi points, GB29, GB30	(concentration) 20,000:1; increase when necessary (dose) 0.2 cc; gradual increase (frequency) 2 sessions/week
	(Phalangeal joint) Ashi points, LI4, LI5, SI4, SI5, TE3, TE5	
Facial nerve palsy [37]	(Idiopathic facial palsy) GB14, LI20, SI18, ST4, ST6, TE17	NS
Gout [41]	Ashi points	NS
Knee osteoarthritis [27]	NS	(dose) NS; but caution advised (frequency) NS; but caution advised
Lumbar herniated intervertebral disk [28]	Ashi, EX-B2, GV points	(concentration) NS; but caution advised (dose) NS; but caution advised
Postoperative syndrome [34]	(Spinal disorder) BL points, EX-B2	(dose) NS; but caution advised (frequency) NS; but caution advised
	(Total knee arthroplasty) NS	
	(Rotator cuff surgery) Ashi points, GB21, LI15, SI11, SI14, SI15, ST12, TE14	
Prostatic hypertrophy [40]	BL31, BL32, BL33, BL34, CV1, CV2, CV3, CV4	NS
Rheumatoid arthritis [31]	Ashi and distal points	NS
Shoulder pain [26]	NS	NS
Stroke [36]	Ashi points, EX-UE70, GB21, LI11, LI15, SI10, SI3, TE14	(Motor disability and spasticity) (dose) 0.05~1 cc (frequency) 12 sessions (duration) 4 w
		(Shoulder pain) (dose) 0.05~0.2 cc (frequency) 3 sessions/week (duration) 2–4 w
Temporomandibular joint disorder [33]	ST6, ST7, TE17, TE21	NS
Tension-type headache [38]	NS	NS
Traffic accident injury (whiplash-associated disorders I, II) [35]	(Neck pain) Ashi points, EX-B2, GB20, GB21, GV14, GV16	NS

* Sweet BVA is recommended for pruritus reduction. Note. Skin pre-tests to identify BV hypersensitivity are recommended in most CPGs. Abbreviations: BL, bladder meridian; BVA, bee venom acupuncture; CPG, clinical practice guideline; CV, conception vessel meridian; EX, extra point; GB, gallbladder meridian; GV, governing vessel meridian; KI, kidney meridian; LI, large intestine meridian; LR, liver meridian; NS, not specified; SI, small intestine meridian; SP, spleen meridian; ST, stomach meridian; TE, triple energizer meridian.

**Table 3:** Evidence of recommendations based on randomized controlled trials for bee venom acupuncture therapy in clinical practice guidelines of traditional Korean medicine.

CPG for	No. of RCTs	Participants Included in the Meta-Analysis	**Evidence Level**	**Reasons for Downgrading**
Standalone therapy				
Chronic low back pain	3	|||||||||||||||||||||||||||||||||||||||||||||||||||||||||||||||||||||||||||||||||||||| (86)	**Moderate**	RoB
Temporomandibular joint disorder	2	|||||||||||||||||||||||||||||||||||||||||||||||||||||||||||||||||||||||||||||||||| (82)	**Moderate**	Imprecision
Knee osteoarthritis	1	||||||||||||||||||||||||||||||||||||||||||||||||||||||||||||||||||||||||||||||||||||||||||||||||||||||||||||||||||||||||||||||||||||||||||||||||||||||||||||||||||||||||||||||||||||||||||||||||||||||||||||||||||||||||||||||||||||||||||||||||||||||||||||||||||||||||||||||||||||||||||||||||||||||||||||||||||||||||||||||||||||||||||||||||||||||||||||||||||||||||||||||| (367)	**Low**	RoB, Imprecision
Rheumatoid arthritis	3	||||||||||||||||||||||||||||||||||||||||||||||||||||||||||||||||||||||||||||||||||||||||||||||||||||||||||||||||||||||||||||||||||||||||||||||||||||||||||||||||||||||||||||||||||||||||||||||||||||||||||||||||||||||||||||||||||||| (229)	**Low**	RoB, Imprecision
Facial nerve palsy	2	||||||||||||||||||||||||||||||||||||||||||||||||||||||||||||||||||||||||||||||||||||||||||||||||||||||||||||||||||||||||||||||||||||||||||||||||| (145)	**Low**	RoB, Imprecision
Combined therapy				
Lumbar herniated intervertebral disk	8	|||||||||||||||||||||||||||||||||||||||||||||||||||||||||||||||||||||||||||||||||||||||||||||||||||||||||||||||||||||||||||||||||||||||||||||||||||||||||||||||||||||||||||||||||||||||||||||||||||||||||||||||||||||||||||||||||||||||||||||||||||||||||||||||||||||||||||||||||||||||||||||||||||||||||||||||||||||||||||||||||||||||||||||||||||||||||||||||||||||||||||||||||||||||||||||| (382)	**Moderate**	Imprecision
Traffic accident injury	3	||||||||||||||||||||||||||||||||||||||||||||||||||||||||||||||||||||||||||||||||||||||||||||||||||||||||||||||||||| (115)	**Moderate**	Imprecision
Shoulder pain	2	||||||||||||||||||||||||||||||||||||||||||||||||||||||||||||||||||||||||||||||||||| (83)	**Moderate**	Imprecision
Cervical pain	2	||||||||||||||||||||||||||||||||||||||||||||||||||||||| (55)	**Moderate**	Imprecision
Cancer-accompanying symptoms	3	|||||||||||||||||||||||||||||||||||||||||||||||||||||||||||||||||||||||||||||||||||||||||||||||||||||||||||||||||||||||||||||||||||||||||||||||||||||||||||||||||||||||||||||||||||||||||||||||||||| (196)	**Low**	RoB, Inconsistency
Stroke (shoulder pain)	5	||||||||||||||||||||||||||||||||||||||||||||||||||||||||||||||||||||||||||||||||||||||||||||||||||||||||||||||||||||||||||||||||||||||||||||||||||||||||||||||||||||||||||||||||||||||||||||| (189)	**Low**	RoB, Imprecision
Rheumatoid arthritis	3	|||||||||||||||||||||||||||||||||||||||||||||||||||||||||||||||||||||||||||||||||||||||||||||||||||||||||||||||||||||||||||||||||||||||||||||||||||||||||||||||||||||||||||||||||||||||||| (186)	**Low**	RoB, Imprecision
Stroke (motor disability)	3	||||||||||||||||||||||||||||||||||||||||||||||||||||||||||||||||||||||||||||||||||||||||||||||||||||||||||||||||||||||||||||||||||||||||||||||||||||| (149)	**Low**	RoB, Imprecision
Ankle sprain	4	|||||||||||||||||||||||||||||||||||||||||||||||||||||||||||||||||||||||||||||||||||||||||||||||||||||||||||||||||| (114)	**Low**	RoB, Imprecision, Inconsistency
Facial nerve palsy (idiopathic facial palsy with post-auricular pain)	3	|||||||||||||||||||||||||||||||||||||||||||||||||||||||||||||||||||||||||||||||||||||||||| (90)	**Low**	RoB, Imprecision
Stroke (spasticity)	2	||||||||||||||||||||||||||||||||||||||||||||||||||||||||||||||| (63)	**Very low**	RoB, Imprecision
Facial nerve palsy (idiopathic facial palsy)	2	|||||||||||||||||||||||||||||||||||||||||||||||||||||||||||| (60)	**Very low**	RoB, Imprecision, Inconsistency
Tension-type headache	1	|||||||||||||||||||||||||||||||||||||||||||||||||||||||||||| (60)	**Very low**	RoB, Imprecision, Indirectness
Carpal tunnel syndrome	1	|||||||||||||||||||||||||||||||||||||||| (40)	**Very low**	RoB, Imprecision

Note. Levels of evidence are evaluated with moderate when the true effect of the treatment could be significantly different with further research, low when the true effect of the treatment could be substantially different with further research, and very low when the true effect of the treatment diverges significantly from the estimate. Abbreviations: CPG, clinical practice guideline; RCT, randomized controlled trial; RoB, risk of bias

**Table 4:** Recommendations for bee venom acupuncture therapy in clinical practice guidelines of traditional Korean medicine.

CPG for	Grade	Harm	Availability	Medical Cost	Patients’ Preference
Ankle sprain [29]	(Acute) C	Assessed with preceding safety reports	Assessed with professionals’ consensus	NA	NA
	(Chronic) C	Assessed with professionals’ consensus	NA	NA	NA
Cancer-accompanying symptoms [39]	C	Assessed with preceding safety reports	Assessed with the official literature and use statistics	Assessed but inconclusive due to insufficient data	Assessed with professionals’ consensus
Carpal tunnel syndrome [30]	C *	Assessed but inconclusive due to insufficient data	Assessed with professionals’ consensus	Assessed but inconclusive due to insufficient data	NA
Cervical pain [25]	B	Assessed with preceding safety reports	NA	NA	NA
Chronic low back pain * [24]	B	Assessed with preceding safety reports	Assessed with professionals’ consensus	Assessed but inconclusive due to insufficient data	NA
Degenerative arthritis [32]	(Hip joint) GPP	Assessed with professionals’ consensus	Assessed with use statistics	NA	NA
	(Phalangeal joint) GPP	Assessed with professionals’ consensus	Assessed with use statistics	NA	NA
Facial nerve palsy [37]	(Facial palsy with delayed treatment) C	Assessed with professionals’ consensus	Assessed with professionals’ consensus	NA	NA
	(Idiopathic facial palsy) C	Assessed with professionals’ consensus	NA	NA	NA
	(Idiopathic facial palsy with post-auricular pain) B *	Assessed but inconclusive due to insufficient data	Assessed with professionals’ consensus	NA	NA
Gout [41]	GPP *	NA	Assessed with use statistics	NA	NA
Knee osteoarthritis [27]	B	Assessed	NA	NA	NA
Lumbar herniated intervertebral disk [28]	B *	Assessed with preceding safety reports	Assessed with use statistics	NA	NA
Postoperative syndrome [34]	C *	Assessed with preceding safety reports	Assessed with professionals’ consensus	Assessed but inconclusive due to insufficient data	NA
Prostatic hypertrophy [40]	GPP *	Assessed, but no specific reasons	Assessed with use statistics	Assessed, but no specific reasons	Assessed, but no specific reasons
Rheumatoid arthritis [31]	C	Assessed with preceding safety reports	Assessed with use statistics	Assessed, but no specific reasons	NA
Shoulder pain [26]	B	Assessed with preceding safety reports	Assessed with professionals’ consensus	NA	NA
Stroke [36]	C	Assessed but inconclusive due to insufficient data	NA	Assessed but inconclusive due to insufficient data	NA
Temporomandibular joint disorder [33]	A *	Assessed with preceding safety reports	Assessed with use statistics	Assessed with economic evaluation	NA
Tension-type headache [38]	C *	Assessed, but no specific reasons	Assessed with the official literature and professionals’ consensus	NA	NA
Traffic accident injury (whiplash-associated disorders I, II) [35]	(Low back and neck pain) C *	Assessed with preceding safety reports	Assessed with professionals’ consensus	NA	NA
	(Neck pain) B *	Assessed	Assessed with use statistics	NA	NA

* BVA therapy is recommended within the scope of pharmacopuncture therapy. Note. Grades of recommendations are assigned with A when the treatment is strongly endorsed for implementation in almost all clinical practices, B when the treatment is advisable in the majority of clinical situations, C when the therapy has potential benefits in some but not all clinical contexts, and D when the treatment is not recommended in most clinical practices. Abbreviations. BVA, bee venom acupuncture; CPG, clinical practice guideline; GPP, good practice point; NA, not assessed.

## Data Availability

The original contributions presented in this study are included in the article/Supplementary Materials. Further inquiries can be directed to the corresponding author.

## Supplementary Materials

The following supporting information can be downloaded at: https://www.mdpi.com/article/10.3390/toxins17040158/s1, Table S1: Recommendations for bee venom acupuncture therapy are included in clinical practice guidelines of traditional Korean medicine.

## Abbreviations

The following abbreviations are used in this manuscript:

AGREE II	Appraisal of Guidelines for Research and Evaluation II
AT	acupuncture therapy
BVA	bee venom acupuncture
BV	bee venom
BL	bladder meridian
CONSORT	Consolidated Standards of Reporting Trials
CPG	clinical practice guideline
CT	cupping therapy
CV	conception vessel meridian
EA	electroacupuncture
EX	extra point
GRADE	Grading of Recommendations, Assessment, Development and Evaluations
GB	gallbladder meridian
GPP	good practice point
GV	governing vessel meridian
HM	herbal medicine
KI	kidney meridian
LI	large intestine meridian
LR	liver meridian
MEL	melittin
MoHW	Ministry of Health and Welfare
NA	not assessed
NCKM	National Clearinghouse for Korean Medicine
NRS	non-randomized studies
NS	not specified
NSAID	non-steroidal anti-inflammatory drug
PT	physical therapy
RoB	risk of bias
RCT	randomized controlled trial
SI	small intestine meridian
SP	spleen meridian
ST	stomach meridian
STRICTA	Standards for Reporting Interventions in Clinical Trials of Acupuncture
TE	triple energizer meridian
TKM	traditional Korean medicine
UC	usual care
