# Supplementary material for: Bee Venom Acupuncture in Traditional Korean Medicine: A Review of Clinical Practice Guidelines
Source: Toxins (Basel). 2025 Mar 22;17(4):158. doi: 10.3390/toxins17040158 (PMC12031355; doi:10.3390/toxins17040158)
Supplement: Supplementary file 1 [file toxins-17-00158-s001.zip › toxins-3523347-supplementary.pdf]

# Bee Venom Acupuncture in Traditional Korean Medicine: A Review of Clinical Practice Guidelines

**Minjung Park and Seungwon Shin**

**Table S1.** Recommendations for bee venom acupuncture therapy included in clinical practice guidelines of traditional Korean medicine.

| CPG for | No | Recomendations | Grade | Evidence Level |
|---|---|---|---|---|
| Ankle sprain | R13 | A combination of acupuncture and BV pharmacopuncture *may be considered* to reduce pain in adult patient with acute ankle sprain. | C | Low |
| | R14 | A combination of acupuncture and BV pharmacopuncture *may be considered* to reduce pain and improve the range of motion in adult patient with chronic ankle sprain. | C | Low |
| Cancer-accompanying symptoms | R9 | Bee-venom therapy, combined with analgesics, *may be considered* to improve cancer-related pain, when compared with analgesics alone. | C | Low |
| Carpal tunnel syndrome | R9* | Concurrent pharmacopuncture and electroacupuncture treatment *may be considered* over electroacupuncture monotherapy to improve symptoms and functionality in adult patients with carpal tunnel syndrome. | C | Very low |
| Cervical pain | R20 | Concurrent treatment with BV pharmacopuncture *should be considered* for the alleviation of symptoms in the clinical treatment in adults with neck pain receiving acupuncture treatment. | B | Moderate |
| Chronic low back pain | R13 | BV therapy *should be considered* to improve pain and function for adult chronic nonspecific low back pain. | B | Moderate |
| Degenerative arthritis (hip and hand) | R4 | BV therapy *is recommended* based on the expert group consensus for functional improvement in degenerative arthritis patients of hip joint. | GPP | NA |
| | R10 | The combination of manual acupuncture with electroacupuncture and BV therapy treatment *is recommended* based on the expert group consensus for functional improvement in degenerative arthritis patients of hip joint. | GPP | NA |
| | R16 | BV therapy *is recommended* based on the expert group consensus for functional improvement in degenerative arthritis patients of phalangeal joint. | GPP | NA |
| Facial nerve palsy | R19 | Practicing BV pharmacopuncture *may be considered* for patients with facial palsy whose treatment is | C | Low |

| | | | | |
|---|---|---|---|---|
| | | delayed rather than general acupuncture treatment. | | |
| | R20 | Practicing general Korean medicinal treatments along with the BV pharmacopuncture therapy *may be considered* for patients with idiopathic facial palsy instead of conducting general Korean medicinal treatments alone. | C | Very low |
| | R22* | It *may be considered* to practice general Korean medicinal treatments along with pharmacopuncture for patients with idiopathic facial palsy accompanied by post-auricular pain rather than giving general Korean medicinal treatments alone. | B | Low |
| Gout | R8* | Pharmacopuncture *can be considered* to improve symptoms in adult patients with acute gout. | GPP | CTB |
| Knee osteoarthritis | R17 | BV therapy *should be considered* for knee osteoarthritis patients. | B | Low |
| Lumbar herniated intervertebral disk | R10* | A combination of pharmacopuncture with usual care *should be considered* for improving overall symptoms of the lumbar herniated intervertebral disc in adults | B | Moderate |
| Postoperative syndrome (spinal disorder) | R6* | A concurrent treatment of pharmaco- or BV acupuncture and conventional rehabilitation or an integrative traditional Korean medicine treatment *may be considered* for patients in the postoperative rehabilitation stage or with persistent or recurrent pain. | C | Low |
| | R11* | Integrative Korean medicine treatment such as acupuncture, electro-acupuncture, moxibustion, cupping, pharmacopuncture (BV), Korean herbal medicine, Chuna, and Korean traditional medicine physiotherapy *may be considered* for patients who had received lumbar spine surgery and reported persistent or recurrent postoperative pain. | C | Low |
| Postoperative syndrome (total knee arthroplasty) | R6* | A combination of herbal acupuncture therapy and conventional rehabilitation therapy *may be considered* for the patient. | C | Very low |
| Postoperative syndrome (rotator cuff surgery) | R10* | It *may be considered* to combine pharmacopuncture (bee-venom pharmacopuncture) therapy and the general rehabilitation treatment rather than performing the general rehabilitation treatment alone in rehabilitation-stage patients after the rotator cuff operation. | C | Very low |
| Prostatic hypertrophy | R6* | For adult men diagnosed with benign prostatic hyperplasia without acute urinary retention, pharmacopuncture monotherapy *is recommended* based on the expert group consensus. | GPP | NA |

| | | | | |
|---|---|---|---|---|
| Rheumatoid arthritis | R3 | Pharmacopuncture treatment *may be considered* to improve symptoms in adult patients with rheumatoid arthritis. | C | Low |
| | R10 | Combined treatment with pharmacopuncture and conventional western medicine treatment *may be considered* to improve symptoms in adult patients with rheumatoid arthritis. | C | Low |
| Shoulder pain | R18 | A BV acupuncture for adult shoulder patients *should be considered*. | B | Moderate |
| Stroke | R90 | Pharmacopuncture using bee-venom *may be considered* to improve motor disability in patients with stroke. | C | Low |
| | R91 | Pharmacopuncture using BV *may be considered* to improve shoulder pain in patients with stroke. | C | Low |
| | R92 | Pharmacopuncture using BV *may be considered* to improve spasticity in patients with stroke. | C | Very low |
| Temporomandibular joint disorder | R5* | Consideration of pharmacopuncture treatment *is recommended* in the clinical practice of temporomandibular joint disorder patients for the improvement of symptoms. | A | Moderate |
| Tension-type headache | R12* | Conducting pharmacopuncture along with herbal medicine treatment *may be considered* in tension type headache patients. | C | Very low |
| Traffic accident injury | R4-1* | Pharmacopuncture according to differentiation of syndromes *may be considered* for symptom alleviation of neck pain and low back pain in whiplash-associated disorders I, II adults (19~70 years old) patients compared to acupuncture treatment only. | C | Very low |
| | R4-2* | A combination of pharmacopuncture with usual care according to differentiation of syndromes *should be consi*dered for symptom improvement of neck pain whiplash-associated disorders I, II adults (19~70 years old) patients compared to acupuncture treatment only. | B | Moderate |

* BVA therapy is recommended within a scope of pharmacopuncture therapy.

*Note*. Grades of recommendations are assigned with; A when the treatment is strongly endorsed for implementation in almost all clinical practices; B when the treatment is advisable in the majority of clinical situations; C when the therapy has potential benefits in some but not all clinical contexts; D when the treatment is not recommended in most clinical practices

*Abbreviations*. BVA, bee venom acupuncture; CPG, clinical practice guideline; CTB, classical text-based; GPP, good practice point; NA, not assessed
